# Supplementary material for: FastqPuri: high-performance preprocessing of RNA-seq data
Source: BMC Bioinformatics. 2019 May 3;20:226. doi: 10.1186/s12859-019-2799-0 (PMC6500068; doi:10.1186/s12859-019-2799-0)
Supplement: Supplementary file 2 — Archive of FastqPuri. Archive containing all files needed to install and run FastqPuri v1.0.6. Date stamp March 22, 2019. (GZ 47,819 kb) [file 12859_2019_2799_MOESM2_ESM.gz › FastqPuri-1.0.6/html/init__Qreport_8c.html]

FastqPuri: src/init\_Qreport.c File Reference


FastqPuri


- src

Functions |
Variables

init\_Qreport.c File Reference

Help dialog for Qreport and initialization of the command line arguments.
More...

`#include <getopt.h>`  
`#include <stdio.h>`  
`#include <string.h>`  
`#include <stdlib.h>`  
`#include "init_Qreport.h"`  
`#include "str_manip.h"`  
`#include "config.h"`  
`#include "defines.h"`

Include dependency graph for init\_Qreport.c:

|  |  |
| --- | --- |
| Functions | |
| void | printHelpDialog\_Qreport () |
|  | Function that prints Qreport help dialog when called. |
|  | |
| void | getarg\_Qreport (int argc, char \*\*argv) |
|  | Reads in the arguments passed through the command line to Qreport. and stores them in the global variable par\_QR. |
|  | |

|  |  |
| --- | --- |
| Variables | |
| Iparam\_Qreport | par\_QR |
|  | |

## Detailed Description

Help dialog for Qreport and initialization of the command line arguments.

Author
:   Paula Perez paula.nosp@m.pere.nosp@m.zrubi.nosp@m.o@gm.nosp@m.ail.c.nosp@m.om

Date
:   03.08.2017

## Variable Documentation

## ◆ par\_QR

Iparam\_Qreport par\_QR

Input parameters of Qreport

global variable: input parameters for Qreport


---

Generated on Mon Mar 19 2018 23:42:01 for FastqPuri by  

 1.8.14
